# Chronic Lymphocytic Leukemia Infiltrating in the Brain

**DOI:** 10.7759/cureus.74080

**Published:** 2024-11-20

**Authors:** Lauren M Webb, Saad S Kenderian, Allison M Angeli, Matthew T Howard, Eelco F Wijdicks

**Affiliations:** 1 Neurology, Mayo Clinic, Rochester, USA; 2 Hematology, Mayo Clinic, Rochester, USA; 3 Internal Medicine, Mayo Clinic, Rochester, USA; 4 Hematopathology, Mayo Clinic, Rochester, USA

**Keywords:** brain biopsy, chronic lymphocytic leukemia (cll), encephalopathy, neuro-oncology, radiology

## Abstract

While earlier post-mortem studies show involvement of the central nervous system in 71% of patients with chronic lymphocytic leukemia (CLL), involvement intravitam is rare. A 72-year-old man with untreated, minimally symptomatic CLL developed subacute-onset encephalopathy and presented with severe hyponatremia and stress-induced cardiomyopathy. His initial head computed tomography scan was unremarkable. His mental status did not improve with careful sodium correction. Magnetic resonance imaging of the brain eventually revealed widespread T2 hyperintensities throughout the cerebral hemispheres, brainstem, and cerebellum. A cerebrospinal fluid analysis demonstrated elevated total nucleated cells (31/mcL, 89% lymphocytes), protein of 75 mg/dL, positive human herpesvirus 6 by polymerase chain reaction, and the presence of malignant CD5+ B cells, consistent with CLL. Brain biopsy confirmed direct infiltration of CLL cells in the brain parenchyma. He was started on zanubrutinib, which led to clinical and radiologic improvement. His neurologic recovery remained slow, and his family elected to transition to comfort-focused care. Our patient’s case exemplifies a rare neurologic manifestation affecting <1% of patients with CLL. Despite partial clinical and radiologic response to zanubrutinib, he had a poor outcome, likely due to the extensive brain areas involved by CLL.

## Introduction

Chronic lymphocytic leukemia (CLL) is the most common adult leukemia in the United States and Europe [1], with a lifetime risk of one in 175 [2]. This lymphoproliferative disorder is characterized by the accumulation of small, monomorphic, mature, clonal B lymphocytes that are CD19-positive with the co-expression of CD5 and CD23 and low expression of CD20 [3]. The spread of CLL cells beyond the peripheral blood, bone marrow, and lymphoid organs is rare [4,5]. CLL usually affects older adults with a median age at diagnosis of 72 years [6]. The etiology of CLL is unknown, but the strongest known risk factor is a family history of hematologic malignancy [3]. Although typically indolent, the aggressiveness of CLL progression is variable [7].

Symptomatic central nervous system (CNS) involvement by CLL is extremely rare, reported in less than 1% of CLL patients [8-10]. We present the case of a 72-year-old male with CLL directly infiltrating the CNS.

## Case presentation

A 72-year-old man presented to the emergency department with two weeks of poor oral intake and progressive confusion. His medical comorbidities included CLL on observation, hypertension, and hyperlipidemia. His CLL was diagnosed two years prior, and he was asymptomatic. He reported an unintentional 5 kg weight loss over six months.

In the emergency department, the patient was afebrile and mildly hypertensive. Physical examination revealed dry mucous membranes and lower extremity edema. He had no meningeal signs. On neurologic exam, he was oriented to self and location but was unable to provide a reliable history. He was noted to have right-sided facial droop, equal strength throughout the arms and legs, and gait instability. Laboratory workup showed a leukocytosis of 42.7 x 10^9^/L (baseline 15-20 x 10^9^/L) and hyponatremia with a sodium of 118 mmol/L. His electrocardiogram showed ST elevations in the anterolateral leads and T wave inversions. The troponin T trend was stably elevated (138, 129, and 134 ng/L). 

**Figure 1:**
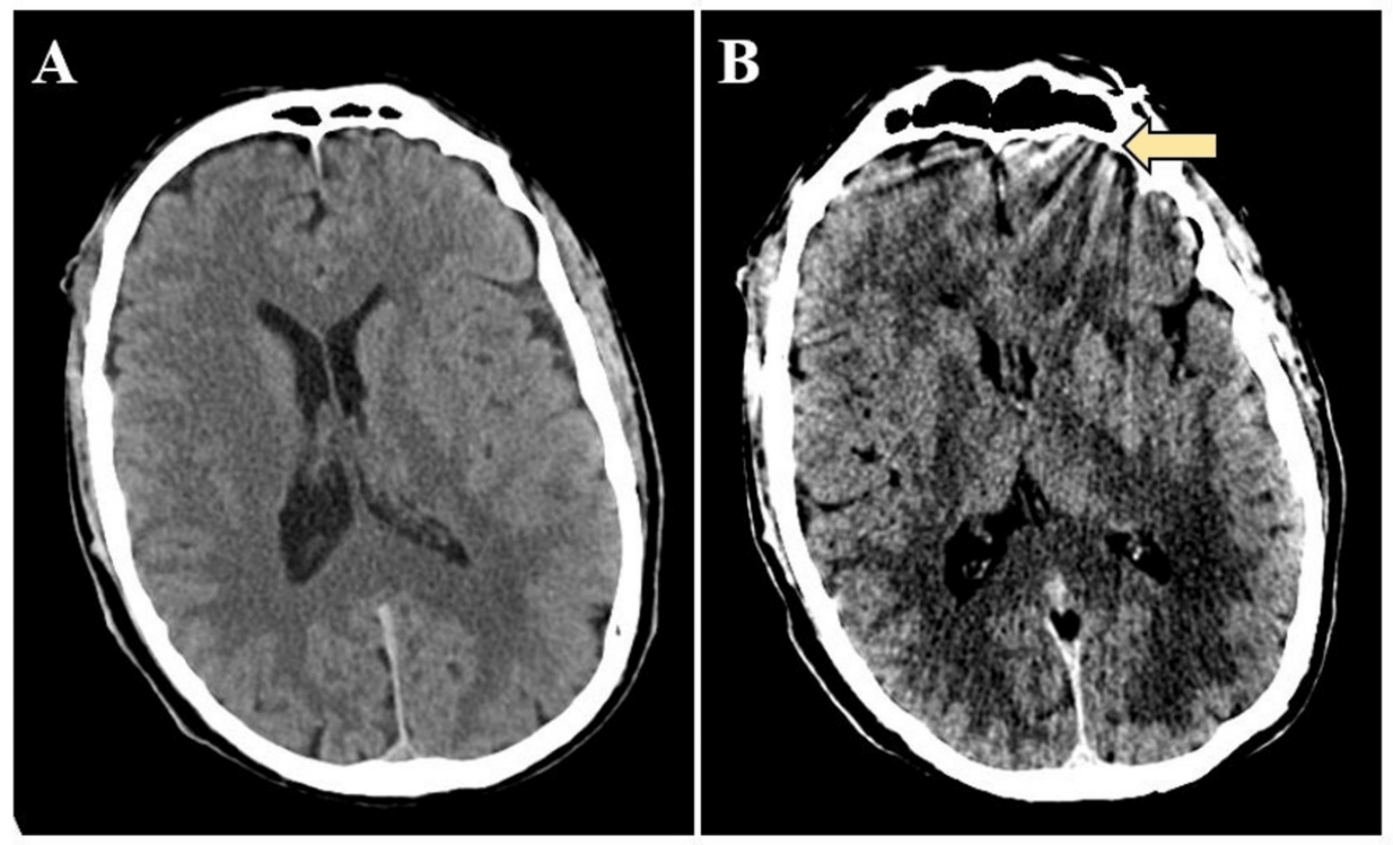
Initial head CT scan A) Head CT scan without IV contrast demonstrating no acute intracranial abnormality. B) Head CT scan showing left frontal metal artifact (yellow arrow).

His CT scan of the head showed a left frontal metal artifact but no other obvious findings (Figure 1). CT angiogram of the head/neck showed patent vasculature and enlarged cervical lymph nodes. A bedside echocardiogram revealed left ventricular enlargement and apical ballooning, concerning for stress-induced cardiomyopathy. He was subsequently admitted to the cardiac intensive care unit for management of stress-induced cardiomyopathy.

The patient's severe hyponatremia was gradually corrected with hypertonic saline over the first three days of his hospitalization. His encephalopathy did not improve with sodium correction.

**Figure 2:**
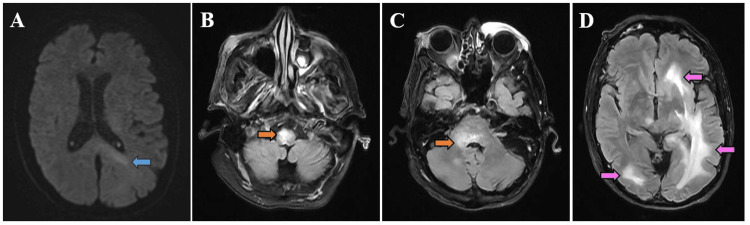
Initial brain MRI A) Diffusion-weighted brain MRI with increased signal in the splenium of the corpus callosum on the left (blue arrow). B-D) FLAIR sequences demonstrating patchy T2 hyperintensities in the brainstem (orange arrows) and cerebral white matter (pink arrows). There was no associated contrast enhancement.

On hospital day 4, the otolaryngology team removed the metallic pellet from the left supraorbital area to allow for MRI. His brain MRI was markedly abnormal with a patchy T2 hyperintense signal involving the bilateral cerebral hemispheres, brainstem, and right cerebellum, causing 3 mm of midline shift (Figure 2). A lumbar puncture performed the same day revealed lymphocytic pleocytosis with 31 total nucleated cells/mcL (89% lymphocytes), protein of 75 mg/dL, and glucose of 75 mg/dL (serum glucose 148 mg/dL). Cerebrospinal fluid (CSF) flow cytometry was positive for malignant CD5+ B cells, consistent with CLL. CSF infectious studies resulted in positive for human herpesvirus 6 (HHV-6) polymerase chain reaction (PCR) and Epstein-Barr virus PCR.

**Figure 3:**
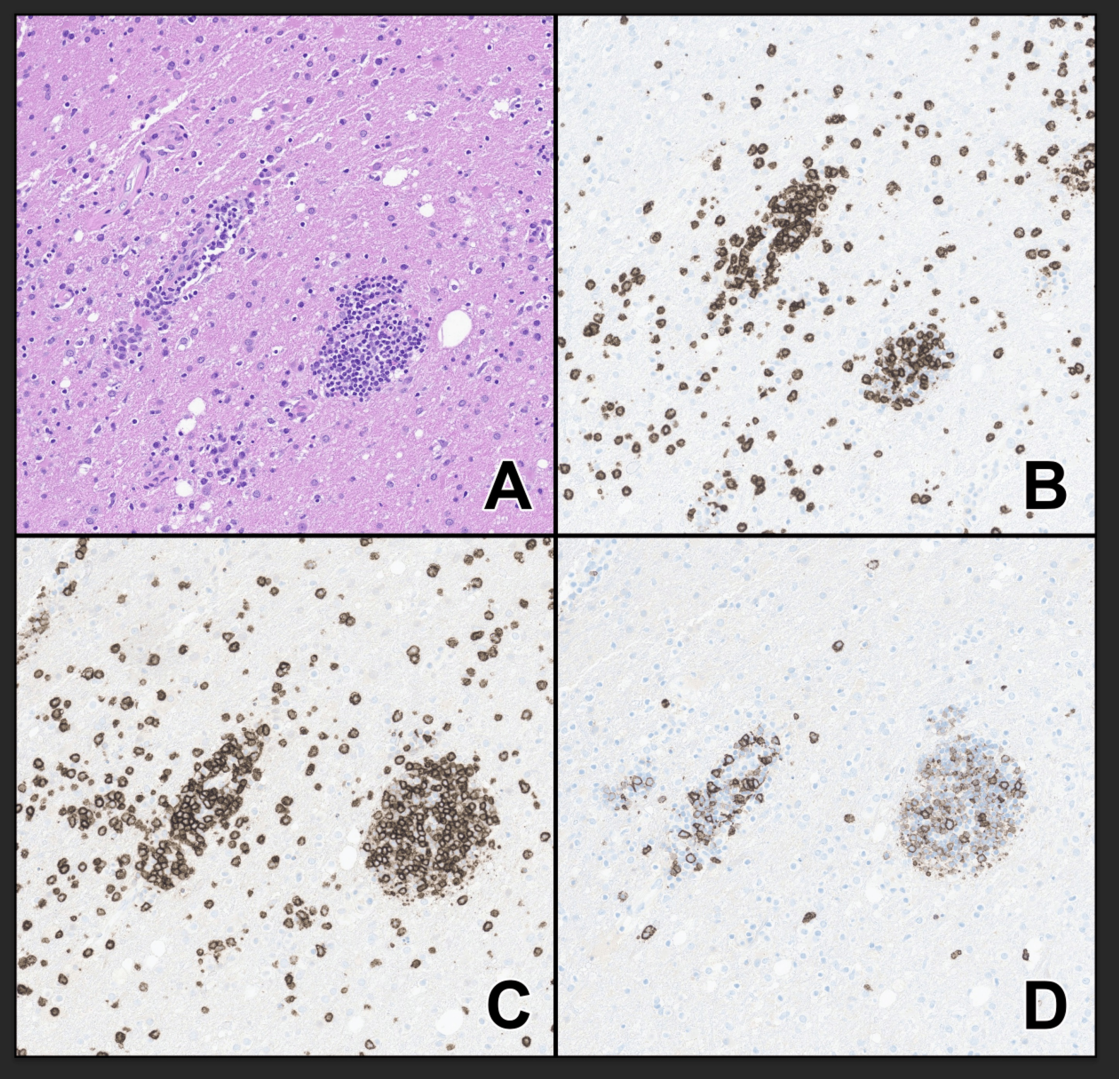
Left parietal lobe brain biopsy pathology A) Hematoxylin and eosin-stained section of the left parietal lobe brain biopsy shows a predominantly perivascular infiltrate of small lymphocytes. B) CD3 stain demonstrating total T-cells. C) CD5 stain showing T-cells that express CD5 and B-cells of CLL, which also express CD5. D) CD20 stain demonstrating B-cells, which characteristically have lower expression of CD20 compared to normal B-cells.  All images were taken at 200x magnification. Concurrently performed flow cytometry identified a kappa light chain restricted B-cell population with the expression of CD5 and CD23.

We initiated treatment with ganciclovir given the possibility of HHV-6 encephalitis. By hospital day 8, the patient's alertness continued to decline, leading to respiratory failure and intubation. On hospital day 9, to clarify his diagnosis, he underwent a stereotactic needle biopsy of the left parietal lobe. The preliminary pathology was consistent with lymphoma, after which he commenced five days of 1g intravenous methylprednisolone. The final brain pathology confirmed perivascular CLL infiltrates consisting of mildly positive CD20 B cells with a weak co-expression of PAX5, CD5, and CD23 (Figure 3), without high-grade transformation or infection. We then initiated CLL treatment with Bruton tyrosine kinase (BTK) inhibitor, zanubrutinib, 160 mg twice daily.

On zanubrutinib, the patient’s alertness and ability to follow commands improved. However, three days after starting zanubrutinib, he developed hematochezia, requiring two red blood cell transfusions and superior rectal artery embolization. His zanubrutinib was held and his alertness and mental status declined. Five days later, zanubrutinib was reinitiated at half dose for three days before increasing to full dose.

**Figure 4:**
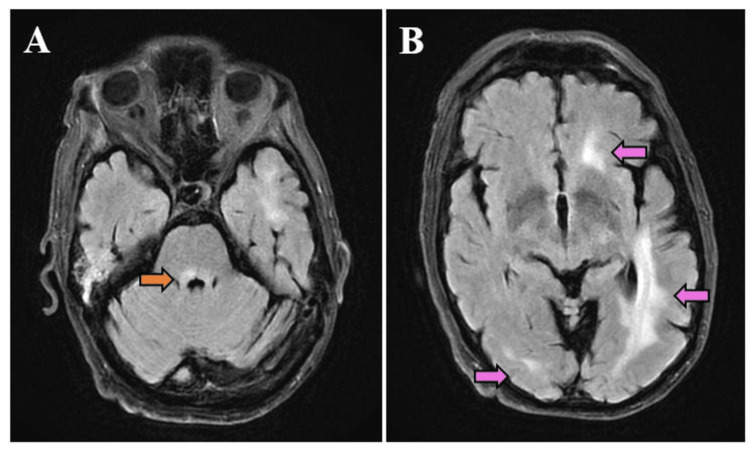
Follow-up brain MRI Fluid-attenuated inversion recovery (FLAIR) magnetic resonance imaging (MRI) obtained after zanubrutinib treatment showing improvement in T2 hyperintensities in the brainstem (A, orange arrow) and cerebral hemispheres (B, pink arrows).

Ganciclovir was discontinued on hospital day 17, after quantitative chromosomal testing revealed chromosomal integration of HHV-6, suggesting his positive HHV-6 PCR in the CSF did not indicate active CNS infection. He remained minimally communicative and required a tracheostomy for ongoing respiratory failure. A repeat brain MRI on hospital day 44 revealed decreased T2 hyperintensities (Figure 4). Considering his slow recovery, long hospitalization, and uncertain neurologic prognosis, his family elected to transition to comfort-focused care, and on hospital day 54, he passed away.

## Discussion

Our patient developed an extremely rare complication of neurologic symptoms from direct brain infiltration by CLL. However, autopsies reveal CLL cells in the CNS in up to 71% of CLL patients, suggesting sub-clinical involvement in most patients [11]. Symptomatic CNS spread can occur at any time in the disease course (median latency is 2.6 years after CLL diagnosis) [12], and as in our patient, it does not correlate with the progression of the CLL disease stage [13,14]. A retrospective analysis of 78 CLL patients with symptomatic CNS involvement found no relationship between the development of neurologic symptoms and gender, age, disease duration, or Rai stage at diagnosis [12,15]. High-risk CLL types have not been linked to increased neurologic symptoms. Our patient had trisomy 12 CLL, associated with an intermediate prognosis [16]. The pathophysiology of CLL spread to the brain is unknown but may involve seeding of the leptomeninges [8]. Insults to the blood-brain barrier like infection or radiation are other potential mechanisms of spread [9,15].

The clinical presentation of CLL invasion of the CNS is highly variable, depending on the location of brain and/or spinal cord involvement. Virtually, any neurologic symptom is possible, including cranial nerve palsies, headaches, vision changes, ataxia, and weakness [8,15]. Symptoms may arise from parenchymal irritation by CLL cells, seizures, local mass effect, and/or elevated intracranial pressure from obstructive hydrocephalus [15]. Our patient’s first sign was encephalopathy, an uncommon manifestation affecting only 15% of patients with symptomatic CNS CLL [8].

Diagnosis is often delayed and confounded, as simultaneous, more common explanations for neurologic symptoms are typically present. Our patient had severe hyponatremia, which developed from the syndrome of inappropriate antidiuretic hormone secretion [17]. His stress-induced cardiomyopathy may have developed secondary to his severe hyponatremia [18].

While there are no formal diagnostic guidelines for CLL involving the CNS, the evaluation includes CSF analysis and MRI, which is more sensitive than a CT scan for CLL [15]. A retrospective study of patients with CNS CLL reported an abnormal CT or MRI in only 32/80 patients [12]. Although our patient’s head CT was unrevealing, his brain MRI showed diffuse white matter hyperintensities, the most frequently observed radiologic abnormality of CNS CLL [10]. The brain MRI provided a target for brain biopsy and served as a baseline for comparison once he started treatment.

Flow cytometry confirmed CLL cells present in our patient’s CSF; however, we proceeded with a brain biopsy due to diagnostic uncertainty and concerns for infection or transformed lymphoma. One retrospective study of 30 patients with symptomatic CNS CLL reported that all patients eventually had positive monoclonal B cells in the CSF, although most patients required two to three lumbar punctures [10].

The positive CSF HHV-6 PCR was a diagnostic distractor. PCR positivity does not definitively imply encephalitis, as HHV-6 can be positive in asymptomatic viral reactivation or viral chromosomal integration, which occurs in approximately 1% of the general population [19]. We initially considered HHV-6 encephalitis a possibility because all CLL patients have some degree of immune system dysfunction [20]. However, his brain MRI did not show limbic involvement typical of HHV-6 encephalitis. His lack of rapid improvement with ganciclovir also argued against HHV-6 encephalitis [19]. Ultimately, serum testing revealed he had chromosomally integrated HHV-6, not indicative of encephalitis.

With a poor neurologic exam and radiologic evidence of mass effect, after we ruled out infection, we administered high-dose corticosteroids, without neurologic improvement. There are no treatment guidelines for CNS CLL, but intrathecal methotrexate, cytarabine, ibrutinib, rituximab, and venetoclax can resolve neurologic symptoms and clear the CSF of CLL cells in some cases [15]. For our patient, we chose zanubrutinib, an oral, potent BTK inhibitor [21] with CSF penetration [10,22]. Zanubrutinib led to clinical improvement within days. Unfortunately, our patient developed gastrointestinal hemorrhage likely from platelet dysfunction, a side effect of zanubrutinib. Our patient also showed marked radiologic improvement after a few weeks of zanubrutinib.

CLL involving the CNS portends a poor prognosis [8,15] with a median overall survival of nine to 10 months [23]. However, over 70% of patients experience clinical improvement with treatment [23] and median overall survival improves with clearance of CLL from the CSF. One retrospective study reported that 90% of patients had a complete or partial hematologic response to treatment and that the five-year overall survival was 72% in patients who were treatment-naïve prior to CNS symptoms [10]. 

## Conclusions

Symptomatic infiltration of the CNS by CLL is exceedingly rare and should be in the differential diagnosis for any CLL patient with leukoencephalopathy or any new neurologic sign or symptom. The clinical presentation of CLL involving the CNS is variable depending on the brain structures involved. The diagnosis may be elusive, as alternative explanations for neurologic dysfunction are often present. Brain MRI, CSF analysis, and brain biopsy were all helpful in confirming the diagnosis for our patient. Although symptomatic CNS involvement of CLL carries a poor prognosis, most patients improve with treatment. Zanubrutinib led to both radiologic and clinical improvement for our patient, but his extensive brain parenchymal and brainstem involvement hindered his neurologic recovery.
